# Cerebral Artery Stenosis, Glymphatic Dysfunction, and Cognitive Trajectories: A Longitudinal Cohort Study of Community‐Dwelling Older Adults

**DOI:** 10.1002/cns.71216

**Published:** 2026-10-21

**Authors:** Weizhuang Yuan, Xingyang Niu, Linfang Lan, Xinran Chen

**Affiliations:** ^1^ Department of Neurology The First Affiliated Hospital, Sun Yat‐sen University Guangzhou China; ^2^ Guangdong Provincial Key Laboratory of Diagnosis and Treatment of Major Neurological Diseases Guangzhou China; ^3^ National Key Clinical Department and Key Discipline of Neurology Guangzhou China

**Keywords:** β‐amyloid, cerebral artery stenosis, cognitive function, glymphatic system, intracranial atherosclerosis, multimodal imaging, vascular dementia

## Abstract

**Aims:**

We aimed to investigate the role of glymphatic dysfunction in the association between stenosis and cognition.

**Methods:**

Older adults from a longitudinal cohort underwent magnetic resonance angiography to quantify cerebral artery stenosis burden. Glymphatic function was evaluated using diffusion tensor imaging‐analysis along the perivascular space (DTI‐ALPS) and free water in white matter (FW‐WM). Global cognitive performance was assessed using the Mini‐Mental State Examination (MMSE) and the Clinical Dementia Rating‐Sum of Boxes (CDR‐SB). Mediation analyses examined indirect effects, and longitudinal cognitive outcomes were analyzed using linear mixed‐effects and Cox proportional hazards models.

**Results:**

Among 346 participants, 87 (25.1%) had at least one stenotic cerebral artery. Stenosis burden was associated with a lower DTI‐ALPS index (*β* = −0.04, *p* = 0.003) and higher FW‐WM (*β* = 0.01, *p* = 0.022). Both DTI‐ALPS and FW‐WM significantly mediated the association between stenosis burden and baseline MMSE, accounting for 13.06%–17.19% of the total effect. In longitudinal analyses, participants with stenosis and impaired glymphatic function had a higher risk of CDR‐SB worsening relative to those with stenosis but preserved glymphatic function (hazard ratios 1.35–2.96) and those without stenosis (hazard ratios 3.27–4.70).

**Conclusion:**

These findings suggest that glymphatic dysfunction may contribute to the association between cerebral artery stenosis and cognitive deterioration.

## Graphical abstract

DTI‐ALPS and FW‐WM significantly mediated the association between stenosis burden and baseline MMSE. Participants with both stenosis and impaired glymphatic function showed the most rapid MMSE decline and a higher risk of CDR‐SB worsening than those with stenosis but preserved glymphatic function or those without stenosis.

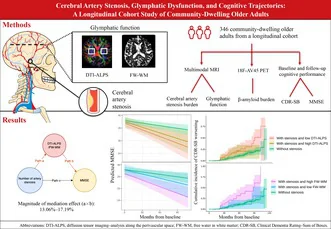

## 1 Introduction

Cognitive impairment and dementia represent a growing public health burden worldwide, with vascular pathology increasingly recognized as a major contributor to late‐life cognitive decline [1, 2]. Cerebral artery stenosis is highly prevalent in aging populations and has been associated with worse cognitive performance and an increased risk of dementia [3, 4, 5, 6]. Previous studies in general populations have reported that the prevalence of asymptomatic intracranial arterial stenosis ranges from 34% to 45% [7]. This condition has been associated with an increased risk of dementia, with hazard ratios (HR) ranging from 1.57 to 1.94 [6].

The mechanisms underlying the association between cerebral artery stenosis and cognitive dysfunction, however, remain incompletely understood. Chronic cerebral hypoperfusion has traditionally been considered the primary pathway, supported by evidence that reduced cerebral blood flow adversely affects neuronal metabolism and network integrity [8, 9, 10]. Nevertheless, hypoperfusion alone does not fully explain the heterogeneity of cognitive outcomes observed among individuals with comparable degrees of arterial stenosis. Moreover, the Atherosclerosis Risk in Communities (ARIC) study revealed that even non‐stenotic plaques (< 50% stenosis), which do not significantly impede blood flow, still increase the risk of cognitive impairment [6]. This suggests that additional downstream mechanisms may play a critical role.

The glymphatic system, a perivascular pathway facilitating cerebrospinal fluid–interstitial fluid exchange and metabolic waste clearance, has emerged as a key regulator of brain homeostasis [11]. Previous studies demonstrate that glymphatic transport depends on arterial pulsatility and intact perivascular spaces, both of which may be compromised in the setting of vascular disease [11, 12, 13]. Accumulating evidence suggests that glymphatic dysfunction is associated with cognitive decline in both normal aging and Alzheimer's disease (AD) [14, 15, 16]. However, direct evidence connecting large‐vessel cerebrovascular pathology to glymphatic impairment in humans remains limited.

Diffusion magnetic resonance (MR) imaging–based metrics provide a noninvasive window into glymphatic function in vivo. The diffusion tensor image analysis along the perivascular space (DTI‐ALPS) index captures water diffusivity aligned with perivascular spaces and has been proposed as a quantitative marker of glymphatic transport [14]. Similarly, increased free water in white matter (FW‐WM) reflects perivascular fluid accumulation and impaired interstitial clearance [14]. Prior studies examining glymphatic function have primarily focused on neurodegenerative diseases [15, 16]. Whether cerebral artery stenosis is associated with impaired glymphatic function, and whether such impairment contributes to accelerated cognitive decline, remain unresolved questions. Moreover, cerebral artery stenosis is closely intertwined with AD pathology. Autopsy studies consistently demonstrate a high burden of atherosclerosis in patients with AD, and vascular dysfunction may exacerbate β‐amyloid (Aβ) accumulation by impairing perivascular clearance pathways [17]. Disentangling the relative contributions of cerebral artery stenosis, glymphatic dysfunction, and Aβ burden to cognitive impairment is therefore essential for clarifying disease mechanisms and identifying potential therapeutic targets.

In this study, we investigated the relationships among cerebral artery stenosis, glymphatic function, and cognition using longitudinal multimodal data from the Open Access Series of Imaging Studies (OASIS‐3) cohort. We assessed whether stenosis is associated with glymphatic dysfunction and whether this dysfunction mediates cognitive impairment. We also examined the relative roles of glymphatic dysfunction and Aβ pathology in cognitive outcomes and determined whether these markers predict an accelerated cognitive decline and clinically meaningful cognitive worsening in individuals with cerebral artery stenosis.

## 2 Methods

### 2.1 Study Population

Data were drawn from the OASIS‐3 cohort [18]. This dataset includes 1378 adults (age 42–95) from the Washington University Knight AD Research Center. Participants underwent longitudinal multimodal imaging and clinical assessments. We included participants who had completed both diffusion‐weighted imaging (DWI) and time‐of‐flight magnetic resonance angiography (TOF‐MRA). To ensure data consistency, participants with single‐shell DWI scans were excluded, as different acquisition parameters preclude direct comparison between them. Individuals with poor‐quality DWI or TOF‐MRA data were also excluded. Cognitive testing, Aβ positron emission tomography (PET), demographic and APOE genotype data were also available. All participants provided written informed consent and the original study was conducted in accordance with the Declaration of Helsinki and approved by the Institutional Review Board of Washington University School of Medicine.

### 2.2 Image Acquisition

All neuroimaging data were obtained using 3T MRI scanners as part of the OASIS‐3 cohort. Diffusion‐weighted images were acquired using a multi‐direction, multi‐*b*‐value protocol ranging from 0 to 1400 s/mm^2^. To ensure methodological consistency, both FW estimation and DTI fitting were restricted to diffusion‐weighted volumes acquired at identical *b*‐values across scans. After harmonization, the final dataset included diffusion images at the following *b*‐values (one direction per *b*‐value): *b* = 0, 50, 100, 150, 200, 300, 350, 400, 450, 500, 600, 700, 800, 850, 900, 950, 1000, 1050, 1100, 1150, 1300, 1350, and 1400 s/mm^2^. Free‐water maps were estimated using the harmonized multi‐*b*‐value dataset described above. For conventional DTI analysis, tensor fitting was performed using diffusion‐weighted images with *b* ≤ 1000 s/mm^2^, while higher *b*‐value images (*b* > 1000 s/mm^2^) were excluded to preserve the assumptions of Gaussian diffusion underlying the tensor model. All MRI data were acquired using Siemens 3T scanners, including a Siemens MAGNETOM TIM Trio and a Siemens Biograph mMR PET‐MR system. The 18F‐AV45 (florbetapir) PET was acquired using a Siemens PET/computed tomography or PET/MR scanner. The imaging standard protocols provided within the OASIS‐3 framework with detailed imaging parameters of DWI, T1, fluid‐attenuated inversion recovery (FLAIR), TOF‐MRA, and PET imaging were previously described [18].

### 2.3 Image Analysis

TOF‐MRA source and maximum‐intensity‐projection images were inspected for stenosis in major intracranial arteries including extracranial (C1–C5, from the proximal internal carotid artery) and intracranial internal carotid artery (C6–C7), anterior cerebral artery (A1/A2), middle cerebral artery (M1/M2), posterior cerebral artery (P1/P2), basilar artery, and vertebral artery. Intracranial arterial stenosis was measured according to the Warfarin–Aspirin Symptomatic Intracranial Disease (WASID) criteria [19]. Extracranial arterial stenosis was measured according to the North American Symptomatic Carotid Endarterectomy Trial (NASCET) method [20]. Prior studies have applied different thresholds to define stenosis, most commonly 70%, 50%, or 30% [21, 22, 23, 24, 25]. In the present study, we adopted a ≥ 30% cutoff to enhance sensitivity for detecting early atherosclerotic lesions, consistent with several population‐based and clinical investigations [23, 24, 25]. This threshold also ensures a sufficient sample size of stenosis‐positive participants to achieve adequate statistical power. A cutoff of ≥ 50% stenosis was used as a sensitivity analysis. To quantify the overall burden of cerebral artery stenosis, we calculated the total number of stenotic arteries among 13 evaluated intracranial and extracranial vessels. Both a binary variable (presence of any stenosis vs. none) and an ordinal variable (number of stenotic vessels) were generated for subsequent analyses. A complete circle of Willis was defined by the presence of all of the vessels that constitute the circle of Willis [26]. All imaging data were independently reviewed by two neurologists (W.Z.Y. and X.Y.N., each with 8 years of experience), who were blinded to the clinical information, using offline commercial software (OsiriX MD v.9.02, Pixmeo SARL, Bernex, Switzerland). If the opinions differed, a third neurologist (L.F.L.) was consulted and the majority opinion prevailed. Given that the stenosis threshold in the present study differed from that used in our earlier studies [22, 27], reliability analyses were additionally performed. The presence of cerebral artery stenosis in the first 50 randomly ordered participants was to calculate inter‐rater reliability. One of the readers (W.Z.Y.) reassessed those images after a 3‐month interval to calculate intra‐rater agreement. The assessment of cerebral artery stenosis presence demonstrated good inter‐rater reliability (Cohen's kappa = 0.70; 95% confidence interval [CI], 0.49–0.91) and intra‐rater reliability (Cohen's kappa = 0.78; 95% CI, 0.60–0.96). Representative cases are shown in Figure S1.

Figure 1 illustrates the overall imaging workflow of the present study. DTI‐ALPS and FW‐WM were used as imaging markers of glymphatic system function. DWI underwent a preprocessing pipeline using FSL (v6.0) and MRtrix3. Preprocessing steps included thermal noise (MP‐PCA), Gibbs unringing, corrections of susceptibility‐induced distortions, eddy currents and movements, followed by diffusion tensor fitting. The resulting diffusivity maps were subsequently registered to the MNI‐152 standard space. Regions of interest were defined as 2.5 mm diameter spheres in the bilateral superior corona radiata (projection fibers) and superior longitudinal fasciculus (association fibers) using coordinates from previous studies [28]. Diffusivities of *x*‐axis in the area of projection fibers (Dx, proj), *x*‐axis in the area of association fibers (Dx, assoc), *y*‐axis in the area of projection fibers (Dy, proj) and *z*‐axis in the area of association fibers (Dz, assoc) were sampled, and the DTI‐ALPS index for each hemisphere was calculated as $\frac{\text{mean}\left(D_{x,\text{proj}},D_{x,\text{assoc}}\right)}{m\mathrm{ea}n\left(D_{y,\text{proj}},D_{z,\text{assoc}}\right)}$. DTI‐ALPS was defined as the mean of left and right values.

**FIGURE 1:**
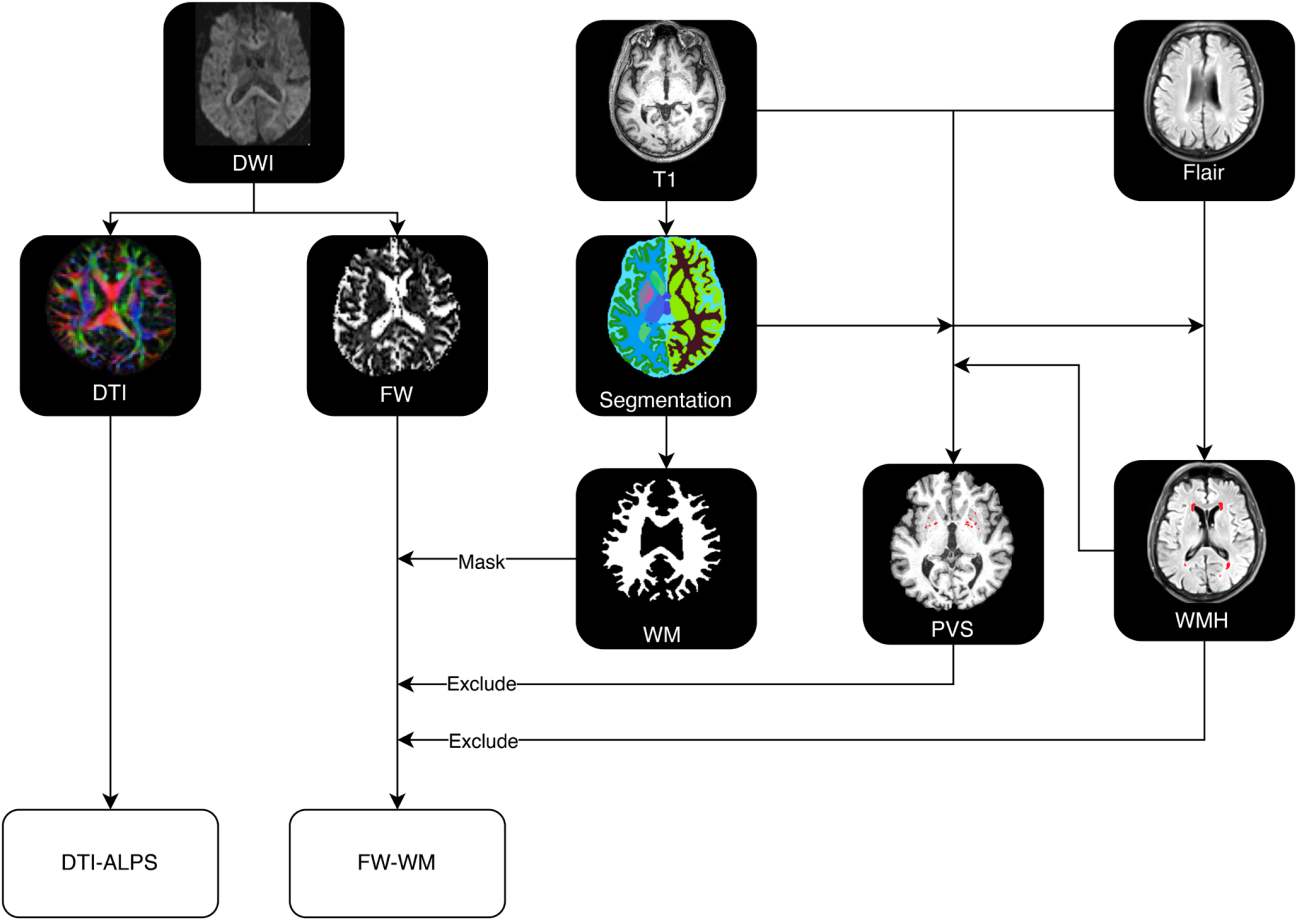
Overview of glymphatic marker analysis. ALPS, analysis along the perivascular space; DTI, diffusion tensor imaging; DWI, diffusion‐weighted imaging; FLAIR, fluid‐attenuated inversion recovery; FW‐WM, free water in white matter; PVS, perivascular space; WM, white matter; WMH, white matter hyperintensity.

FW‐WM was computed using the DIPY library only in participants with contemporaneous T1‐weighted and FLAIR images, which enabled accurate white matter masking and exclusion of white matter hyperintensities and enlarged perivascular spaces. This method models the diffusion signal as a combination of a free water compartment (isotropic diffusion) and a tissue compartment [29]. A white matter mask was generated from T1‐weighted images using SynthSeg, and areas of white matter hyperintensities and enlarged perivascular spaces were excluded to minimize confounding. White matter hyperintensities and enlarged perivascular spaces segmentation were performed using the segcsvd algorithm [30, 31].

18F‐AV45 PET was used to assess brain Aβ burden [32]. Regional standardized uptake value ratio (SUVR) was calculated using cerebellar gray matter as a reference, with partial‐volume correction via a regional spread function approach. Mean cortical SUVR was derived by averaging values from the bilateral lateral and medial orbitofrontal, precuneus, rostral middle frontal, superior frontal, superior temporal, and middle temporal cortices [33]. PET scans performed within 3 months of the baseline MRI were considered baseline data.

### 2.4 Cognitive Assessments

Global cognitive function was assessed using the Mini‐Mental State Examination (MMSE) and the Clinical Dementia Rating‐Sum of Boxes (CDR‐SB). The CDR‐SB was calculated as the sum of scores across all six domains. Baseline cognition was defined as the assessment closest to the imaging date (within 3 months prior or the first post‐imaging cognitive assessment). Longitudinal decline was defined in two ways: (1) MMSE and CDR‐SB scores at each follow‐up visit; (2) time to a cognitive worsening, which we defined as an increase in CDR‐SB of ≥ 1.5 points from baseline. This threshold was selected to represent a meaningful within‐patient change, based on prior studies in individuals with mild cognitive impairment and early AD, which have shown that CDR‐SB increases of approximately 1.0–2.5 points are generally considered clinically relevant [34].

### 2.5 Statistical Analysis

Continuous variables were summarized as mean ± standard deviation or median with interquartile range, and categorical variables were presented as counts and percentages. Group comparisons among the three groups were performed using one‐way ANOVA or Kruskal–Wallis tests for continuous variables and chi‐square tests or Fisher's exact tests for categorical variables, as appropriate. Linear regression models were used to assess the associations between cerebral artery stenosis burden (defined as the number of stenotic arteries) and glymphatic function markers (DTI‐ALPS and FW‐WM). Simple Mediation analyses were performed to investigate whether glymphatic function markers (DTI‐ALPS and FW‐WM) mediated the relationship between cerebral artery stenosis burden and baseline cognitive performance (MMSE and CDR‐SB). Indirect and total effects were estimated using bootstrap resampling with 1000 iterations, and 95% confidence intervals (CIs) were calculated. All mediation models were adjusted for age, sex, years of education, the interval between baseline imaging and cognitive assessment, vascular risk factors (including hypertension, diabetes mellitus, and hyperlipidemia), history of stroke, white matter hyperintensity volume, completeness of the circle of Willis, and scanner model. Additional sensitivity analyses included adjustment for Aβ burden in participants with available baseline Aβ PET data. To further elucidate the role of Aβ pathology in the association between cerebral artery stenosis, glymphatic dysfunction, and cognitive performance, additional multiple mediation analyses were conducted. In these models, cerebral artery stenosis burden was specified as the independent variable, cognitive performance (MMSE or CDR‐SB) as the outcome, and glymphatic function markers (DTI‐ALPS or FW‐WM) and Aβ PET as parallel mediators. The total indirect effect was decomposed into three specific pathways: (1) cerebral artery stenosis → glymphatic dysfunction → Aβ PET → cognitive performance; (2) cerebral artery stenosis → glymphatic dysfunction → cognitive performance; and (3) cerebral artery stenosis → Aβ PET → cognitive performance. All models were adjusted for age, sex, years of education, the interval between baseline imaging and cognitive assessment, vascular risk factors (including hypertension, diabetes mellitus, and hyperlipidemia), history of stroke, white matter hyperintensity volume, completeness of the circle of Willis, and scanner model. Standardized coefficients (*β*) were calculated for each path to allow direct comparison of the relative contribution of individual indirect pathways, thereby identifying the dominant mediating mechanism. Longitudinal cognitive trajectories were analyzed using linear mixed‐effects models with random intercepts and slopes to account for repeated measures within participants. Fixed effects included baseline age, sex, years of education, vascular risk factors (including hypertension, diabetes mellitus, and hyperlipidemia), history of stroke, white matter hyperintensity volume, completeness of the circle of Willis, scanner model, cerebral artery stenosis status, glymphatic function group, and their interactions with time. The primary outcomes were the estimated mean trajectories of MMSE and CDR‐SB scores over time. Time‐to‐event analyses were performed using Kaplan–Meier curves to evaluate progression to cognitive worsening. Cox proportional hazards models were used to estimate HR and 95% CIs, adjusting for baseline age, sex, years of education, scanner model, and baseline cognitive score. Proportional hazards assumptions were verified by Schoenfeld residuals. The robustness of the main findings was evaluated through sensitivity and subgroup analyses. A cutoff of ≥ 50% stenosis was used as a sensitivity analysis. Given distinct anatomical and pathophysiological characteristics of intracranial and extracranial arterial stenosis [7], we performed subgroup analyses by examining intracranial and extracranial stenosis separately, excluding participants with the other type of stenosis from each analysis. These findings should be interpreted with caution as potentially exploratory because no adjustment was made for multiple comparisons. An exploratory post hoc analysis was conducted to compare the correlations of DTI‐ALPS with anterior and posterior circulation stenosis burdens. Stenosis burdens were calculated separately for the anterior and posterior circulations. The two dependent, overlapping correlations were compared using a two‐sided Williams test [35]. The difference in correlations was defined as anterior minus posterior, with a 95% confidence interval calculated [36]. All analyses were two‐sided, with *p* < 0.05 considered statistically significant, and were performed in R (version 4.0.3; R Foundation for Statistical Computing, Vienna, Austria).

## 3 Results

### 3.1 Participants Characteristics

A total of 1378 participants from the OASIS‐3 imaging database were initially screened. Participants without complete DWI or TOF‐MRA data (*n* = 1003), as well as 17 participants with single‐shell DWI scans, were excluded. Among the remaining 358 participants eligible for imaging analysis, two were excluded due to poor‐quality DWI and 10 due to poor TOF‐MRA image quality. The final cohort consisted of 346 participants (Figure S2). The characteristics of included and excluded participants are presented in Table S1. Compared with participants who were not included in the final analysis, the included cohort was younger and had a lower number of strokes or transient ischemic attack occurring at baseline and during follow‐up, while other characteristics were largely comparable between the two groups. Of these, 335 participants completed baseline cognitive assessments. During follow‐up, 291 participants completed at least one follow‐up cognitive assessment, and 244 participants completed two or more follow‐up assessments. At baseline, both MMSE and CDR‐SB assessments were conducted a median of 32 days prior to imaging. The median duration from baseline imaging to the final follow‐up was 4.0 and 5.7 years for MMSE and CDR‐SB, respectively.

Among the 346 participants, 87 participants (25.1%) had at least one stenotic artery, with a total of 155 stenotic arteries identified. The distribution of stenosis was as follows: extracranial internal carotid artery, 23 on the left and 20 on the right; intracranial internal carotid artery, 1 on the left and 2 on the right; middle cerebral artery, 13 on the left and 15 on the right; anterior cerebral artery, 7 on the left and 9 on the right; posterior cerebral artery, 27 on the left and 17 on the right; vertebral artery, 5 on the left and 9 on the right; basilar artery, 7. Additional baseline characteristics are provided in Table S2.

The mean age of the cohort was 66.4 ± 9.1 years, and 189 participants (54.6%) were female. The majority were White (*n* = 294, 85.0%) with a median education level of 16 years (interquartile range, 14–18 years). A history of stroke or transient ischemic attack was reported in 15 participants (4.3%), and 60 participants (17.3%) had a history of AD. The DTI‐ALPS index was calculable for all participants, whereas FW‐WM could not be calculated for 24 participants due to the absence of concurrent FLAIR or T1‐weighted images.

### 3.2 Association of Cerebral Artery Stenosis With Glymphatic Function at Baseline

Figure 2 illustrates the relationship between cerebral artery stenosis and glymphatic function at baseline. Participants with cerebral artery stenosis had a significantly lower DTI‐ALPS index compared with those without stenosis (1.15 ± 0.21 vs. 1.24 ± 0.23; *p* = 0.001, Figure 2A) and a higher FW‐WM (0.22 ± 0.05 vs. 0.21 ± 0.04; *p* = 0.044, Figure 2B). The number of stenotic arteries was negatively correlated with DTI‐ALPS index (*β* = −0.04, *p* = 0.003, Figure 2C) and positively correlated with FW‐WM (*β* = 0.01, *p* = 0.022, Figure 2D). When cerebral artery stenosis was further stratified into 30% to < 50% stenosis and ≥ 50% stenosis, we found a progressive decline in DTI‐ALPS index and a corresponding progressive increase in FW across the no‐stenosis, 30 to < 50% stenosis, and ≥ 50% stenosis groups (Figure S3). However, no significant association was observed between cerebral artery stenosis and Aβ pathology.

**FIGURE 2:**
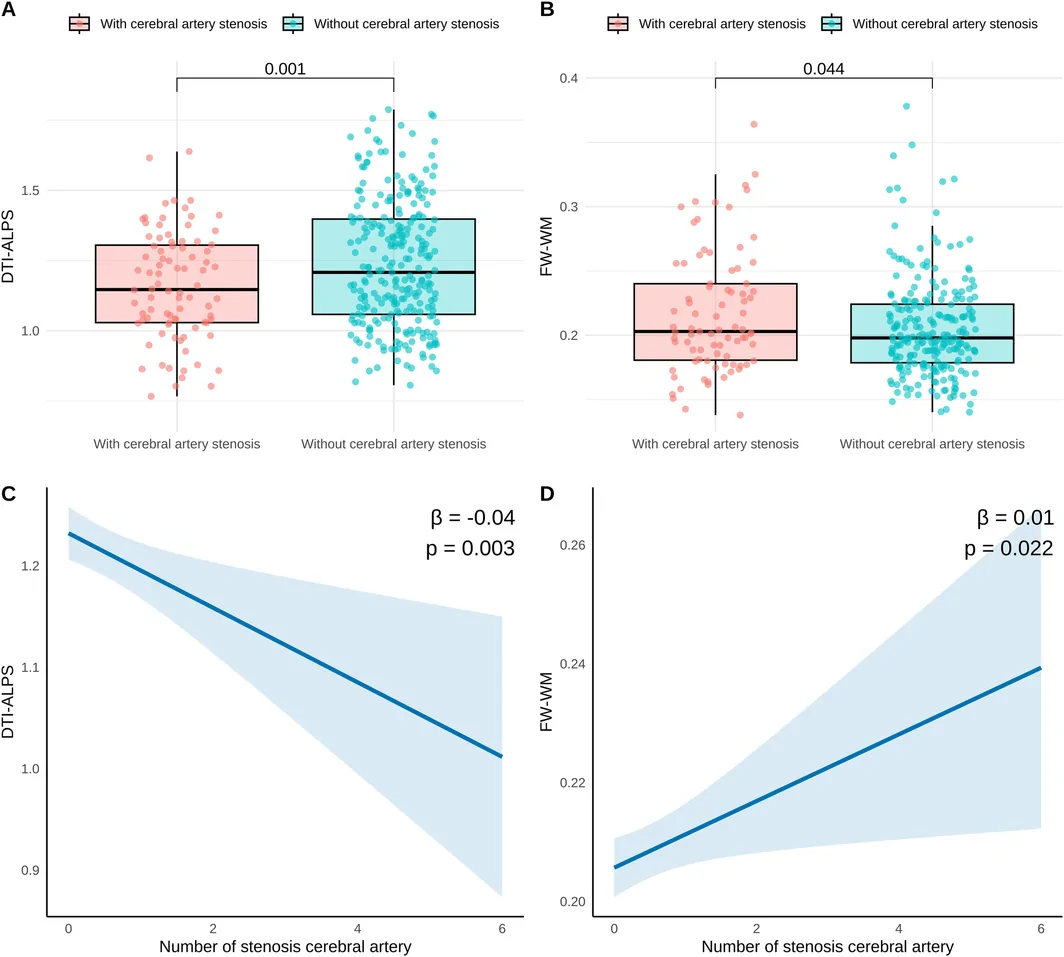
Association of cerebral artery stenosis with glymphatic function. (A) Boxplots comparing the DTI–ALPS index between participants with and without cerebral artery stenosis. (B) Boxplots comparing FW‐WM between participants with and without cerebral artery stenosis. (C) Scatterplot showing the association between the number of stenotic cerebral arteries and the DTI–ALPS index. (D) Scatterplot showing the association between the number of stenotic cerebral arteries and FW‐WM. In C and D, the solid blue line represents the fitted line and the shaded area indicates the 95% confidence interval. DTI–ALPS, diffusivity along the perivascular space index; FW‐WM, white matter free water.

### 3.3 Mediation of Glymphatic Function and Aβ Burden in the Association Between Cerebral Artery Stenosis and Cognitive Performance at Baseline

At baseline, cerebral artery stenosis burden, defined as the number of cerebral stenotic arteries, was significantly associated with worse cognitive performance, including lower MMSE scores (*β* = −0.36; 95% CI –0.57 to −0.14; *p* = 0.001) and higher CDR‐SB scores (*β* = 0.18; 95% CI 0.03 to 0.34; *p* = 0.021). Mediation analyses were then performed to assess whether the DTI‐ALPS index and FW‐WM mediated the association between cerebral artery stenosis and baseline cognitive performance (Figure 3). All models were adjusted for age, sex, years of education, the interval between imaging and baseline cognitive assessment, vascular risk factors (including hypertension, diabetes mellitus, and hyperlipidemia), history of stroke, white matter hyperintensity volume, completeness of the circle of Willis, and scanner model. For MMSE, both DTI‐ALPS and FW‐WM significantly mediated the association between cerebral artery stenosis burden and cognitive performance. The indirect effect through DTI‐ALPS was −0.032 (95% CI –0.074 to −0.004; *p* = 0.032), accounting for 13.06% of the total effect, while the indirect effect through FW‐WM was −0.059 (95% CI –0.144 to −0.003; *p* = 0.034), accounting for 17.19% of the total effect. For CDR‐SB, the indirect effect mediated by DTI‐ALPS was statistically significant (*β* = 0.024; 95% CI, 0.002 to 0.053; *p* = 0.040; accounting for 18.85% of the total effect), whereas the indirect effect mediated by FW‐WM was not reached statistical significance (*β* = 0.043; 95% CI, −0.002 to 0.104; *p* = 0.060; accounting for 21.04% of the total effect).

**FIGURE 3:**
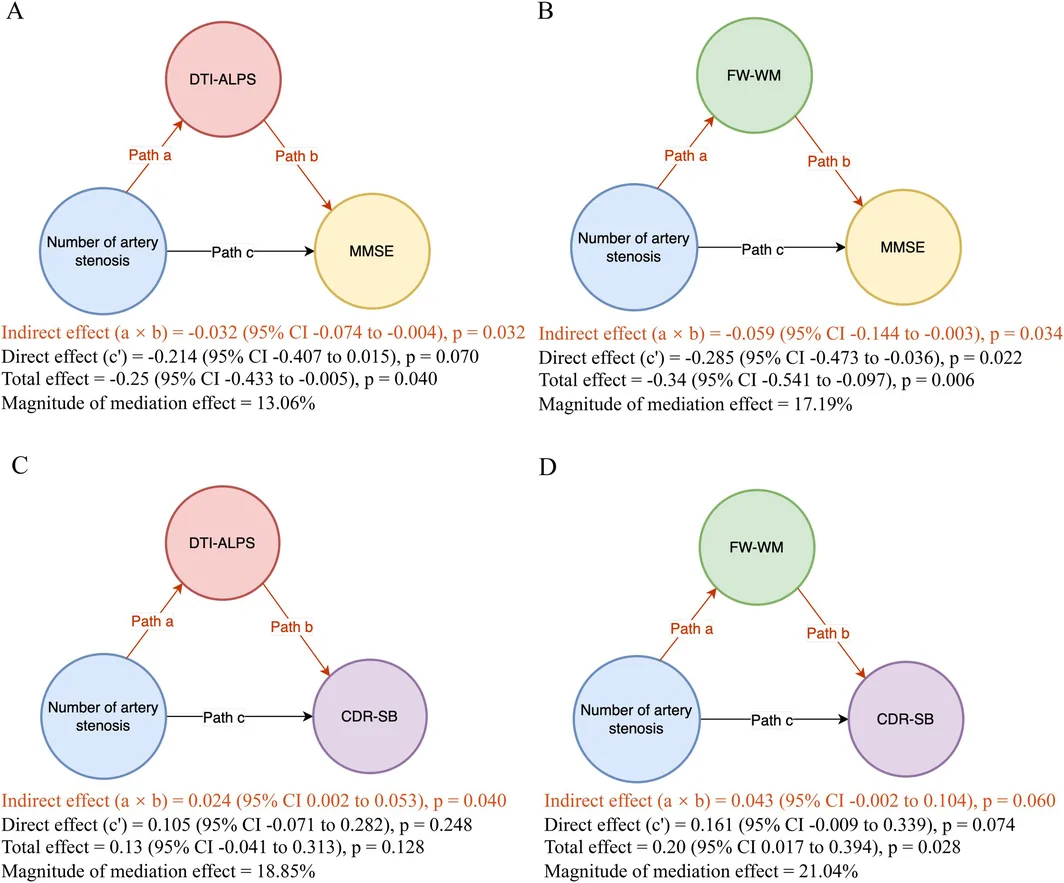
Mediation of the association between cerebral artery stenosis and cognitive performance by glymphatic function at baseline. (A) Indirect effect of the number of cerebral artery stenosis on MMSE mediated by DTI‐ALPS. (B) Indirect effect of the number of cerebral artery stenosis on MMSE mediated by FW‐WM. (C) Indirect effect of the number of cerebral artery stenosis on CDR‐SB mediated by DTI‐ALPS. (D) Indirect effect of the number of cerebral artery stenosis on CDR‐SB mediated by FW‐WM. All models were adjusted for age, sex, years of education, the interval between imaging, baseline cognitive assessment, history of stroke, vascular risk factors (including hypertension, diabetes mellitus, and hyperlipidemia), white matter hyperintensity volume, completeness of the circle of Willis, and scanner model. CDR‐SB, Clinical Dementia Rating–Sum of Boxes; DTI‐ALPS, diffusion tensor imaging–analysis along the perivascular space; FW‐WM, free water in white matter; MMSE, Mini‐Mental State Examination.

Given the close pathophysiologic relationship between cerebral atherosclerosis and AD pathology, additional analyses were conducted in participants with available Aβ PET imaging (*n* = 297), with further adjustment for Aβ burden. The mediation effects of DTI‐ALPS and FW‐WM remained materially unchanged after additional adjustment for Aβ (Figure S4).

To further disentangle the relative contributions of glymphatic dysfunction and Aβ pathology, we constructed multiple mediation models incorporating cerebral artery stenosis burden, Aβ PET, cognitive performance, and glymphatic function markers (Figure 4). In these models, cerebral artery stenosis burden was significantly associated with DTI‐ALPS (*β* = −0.157; *p* = 0.006) but not with Aβ PET (*β* = 0.048; *p* = 0.186). The proportion of the total mediated effect attributable to DTI‐ALPS (64.7%) was substantially greater than that attributable to Aβ PET (29.4%). A similar pattern was observed for FW‐WM in relation to MMSE, although none of the individual indirect pathways reached statistical significance. For CDR‐SB, the overall pattern was comparable, with a relatively greater contribution of Aβ PET to the indirect effect, while glymphatic dysfunction remained a dominant pathway.

**FIGURE 4:**
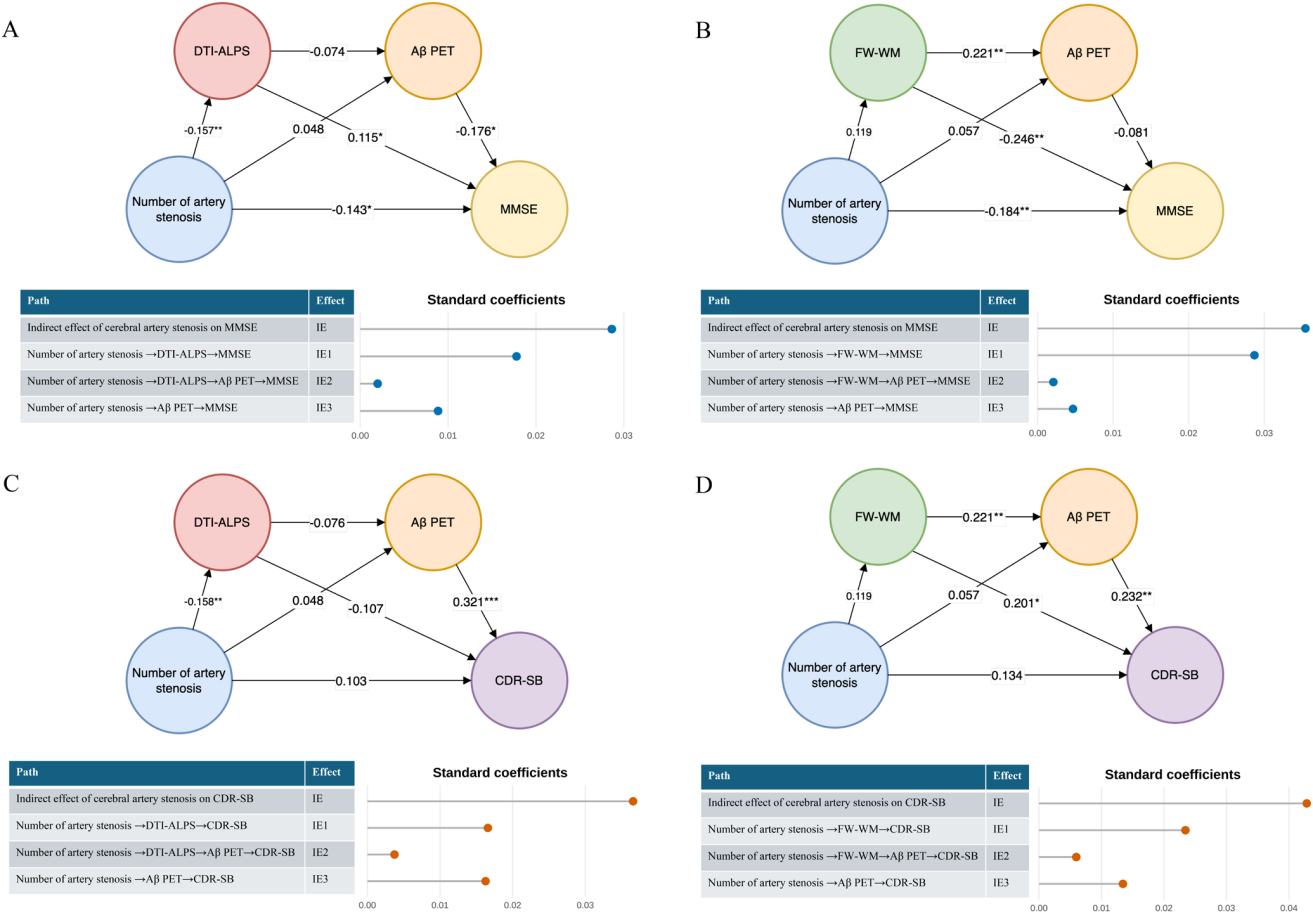
Multiple mediation analysis of the effects of cerebral artery stenosis burden on baseline cognitive performance through glymphatic function markers and Aβ PET. This figure presents the standardized coefficients (*β*) for each pathway in the multiple mediation model, which includes cerebral artery stenosis burden, Aβ PET, cognitive performance, and glymphatic function markers (DTI‐ALPS and FW‐WM). (A, B) Indirect effect of the number of stenotic arteries on the MMSE scores, mediated by (A) DTI‐ALPS and (B) FW‐WM. (C, D) Indirect effect of the number of stenotic arteries on the CDR‐SB scores, mediated by (C) DTI‐ALPS and (D) FW‐WM. *p* values are indicated by asterisks: *p < 0.05, **p < 0.01, ***p < 0.001. All models were adjusted for age, sex, years of education, the interval between imaging and baseline cognitive assessment, history of stroke, vascular risk factors (including hypertension, diabetes mellitus, and hyperlipidemia), white matter hyperintensity volume, completeness of the circle of Willis, and scanner model. In each lollipop plot, the horizontal gray line represents the absolute value of the standardized coefficient (*β*), while the color of the circles denotes the direction of the effect: orange for positive and blue for negative associations. DTI‐ALPS, diffusion tensor imaging‐analysis along the perivascular space; FW‐WM, free water in white matter; MMSE, Mini‐Mental State Examination; CDR‐SB, Clinical Dementia Rating‐Sum of Boxes; Aβ PET, amyloid‐β positron emission tomography.

### 3.4 Participant Classification According to Cerebral Artery Stenosis and Glymphatic Function

Participants were first categorized according to the presence of cerebral artery stenosis into those with stenosis (*n* = 87) and those without stenosis (*n* = 259). Among participants with cerebral artery stenosis, glymphatic function was further stratified using the median DTI‐ALPS value of the entire cohort (1.19), resulting in a low DTI‐ALPS group (*n* = 47) and a high DTI‐ALPS group (*n* = 40). Similarly, FW‐WM was dichotomized using the cohort median value (0.20) into a high FW‐WM group (*n* = 47) and a low FW‐WM group (*n* = 36). Low DTI‐ALPS and high FW‐WM were considered indicative of impaired glymphatic function.

Baseline characteristics of the stratified groups are summarized in Table S3. Participants with cerebral artery stenosis and low DTI‐ALPS were older (71.7 ± 7.6 years) compared with those with stenosis and high DTI‐ALPS (65.12 ± 8.27 years) and those without stenosis (65.6 ± 9.1 years). Similarly, participants with stenosis and high FW‐WM were older than those with stenosis and low FW‐WM (69.7 ± 9.0 vs. 66.72 ± 7.74 years) and those without stenosis (65.6 ± 9.1 years). A higher prevalence of AD was observed among participants with cerebral artery stenosis and impaired glymphatic function. Specifically, AD was more frequent in the low DTI‐ALPS group (16 [34.0%]) compared with the high DTI‐ALPS group (5 [12.5%]) and participants without stenosis (39 [15.1%]). A similar pattern was observed for FW‐WM, with a higher prevalence of AD in participants with high FW‐WM (14 [29.8%]) compared with those with low FW‐WM (6 [16.7%]) and participants without stenosis (39 [15.1%]). Other baseline demographic characteristics, including traditional vascular risk factors, did not differ significantly among groups.

### 3.5 Accelerated Longitudinal Cognitive Decline in Participants With Cerebral Artery Stenosis and Glymphatic Dysfunction

Figure 5 and Table 1 illustrate longitudinal cognitive trajectories across groups stratified by cerebral artery stenosis and glymphatic function. After adjustment for baseline age, sex, years of education, vascular risk factors, white matter hyperintensity volume, completeness of the circle of Willis, and scanner model, participants with cerebral artery stenosis and impaired glymphatic function exhibited the fastest rate of cognitive decline. For MMSE, participants with stenosis and low DTI‐ALPS exhibited the fastest annual decline (−0.42 points/year, 95% CI −0.64 to −0.19), significantly faster than participants without stenosis (between‐group difference −0.26 points/year, 95% CI −0.50 to −0.02; *p* = 0.036) and no significant difference in those with stenosis and high DTI‐ALPS (between‐group difference −0.07 points/year, 95% CI −0.38 to 0.24; *p* = 0.672). Consistent results were observed with FW‐WM stratification, in which participants with stenosis and high FW‐WM showed the fastest MMSE decline (−0.44 points/year, 95% CI −0.65 to −0.24), significantly faster than participants without stenosis (between‐group difference −0.28 points/year, 95% CI −0.50 to −0.07; *p* = 0.011) and did not differ significantly in those with stenosis and low FW‐WM (between‐group difference −0.15 points/year, 95% CI −0.47 to 0.16; *p* = 0.342). For CDR‐SB, participants with stenosis and low DTI‐ALPS demonstrated the fastest annual increase (0.70 points/year, 95% CI 0.47 to 0.94), greater than those without stenosis (between‐group difference 0.50 points/year, 95% CI 0.25 to 0.75; *p* < 0.001) and those with stenosis and high DTI‐ALPS (between‐group difference 0.42 points/year, 95% CI 0.10 to 0.75; *p* = 0.011). Similar findings were observed using FW‐WM stratification, participants with stenosis and high FW‐WM showed the fastest annual increase in CDR‐SB (0.71 points/year, 95% CI 0.48 to 0.93), greater than participants without stenosis (between‐group difference 0.50 points/year, 95% CI 0.26 to 0.74; *p* < 0.001) and in those with stenosis but low FW‐WM (between‐group difference 0.46 points/year, 95% CI 0.12 to 0.80; *p* = 0.008).

**FIGURE 5:**
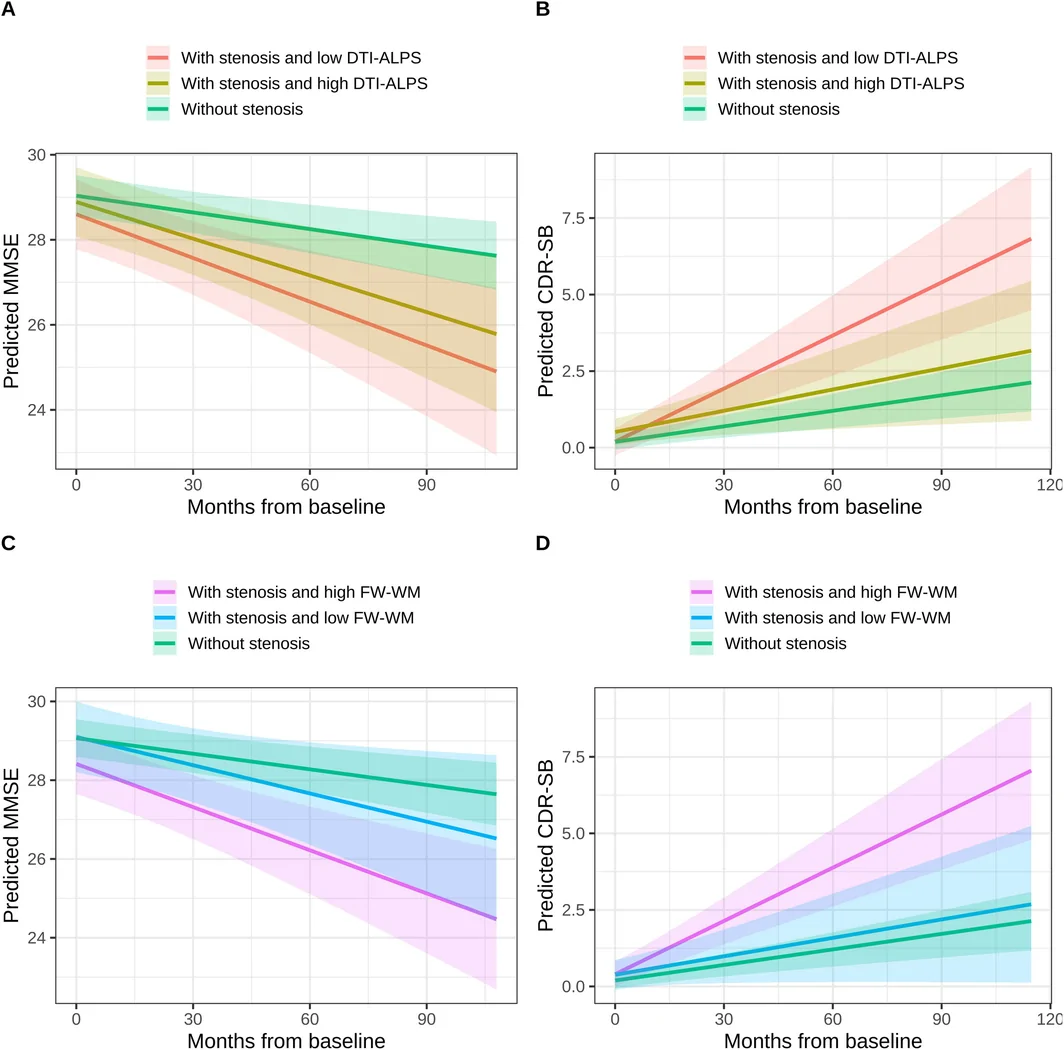
Longitudinal trajectories of cognitive performance by cerebral artery stenosis and glymphatic function groups. (A) Estimated mean MMSE scores over time for participants stratified by stenosis and DTI‐ALPS levels. (B) Estimated mean CDR‐SB scores over time for participants stratified by stenosis and DTI‐ALPS levels. (C) Estimated mean MMSE scores over time for participants stratified by stenosis and FW‐WM levels. (D) Estimated mean CDR‐SB scores over time for participants stratified by stenosis and FW‐WM levels. All models were adjusted for baseline age, sex, years of education, history of stroke, vascular risk factors (including hypertension, diabetes mellitus, and hyperlipidemia), white matter hyperintensity volume, completeness of the circle of Willis, and scanner model. Shaded regions represent 95% confidence intervals. CDR‐SB, Clinical Dementia Rating‐Sum of Boxes; DTI‐ALPS, diffusion tensor imaging‐analysis along the perivascular space; FW‐WM, free water in white matter; MMSE, Mini‐Mental State Examination.

**TABLE 1:** Longitudinal cognitive decline by cerebral artery stenosis and glymphatic function.

Cognitive measure	Group	Slope (95% CI) (points/year)	Compare to without stenosis		Compare to stenosis with preserved glymphatic function	
			Difference in slope (95% CI) (points/year)	*p*	Difference in slope (95% CI) (points/year)	*p*
MMSE	DTI‐ALPS					
	With stenosis and low DTI‐ALPS	−0.42 (−0.64 to −0.19)	−0.26 (−0.50 to −0.02)	0.036	−0.07 (−0.38 to 0.24)	0.672
	With stenosis and high DTI‐ALPS	−0.35 (−0.56 to −0.14)	−0.19 (−0.42 to 0.03)	0.095	—	
	Without stenosis	−0.16 (−0.24 to −0.08)	—		—	
	FW‐WM					
	With stenosis and high FW‐WM	−0.44 (−0.65 to −0.24)	−0.28 (−0.50 to −0.07)	0.011	−0.15 (−0.47 to 0.16)	0.342
	With stenosis and low FW‐WM	−0.29 (−0.54 to −0.05)	−0.13 (−0.39 to 0.13)	0.322	—	
	Without stenosis	−0.16 (−0.24 to −0.08)	—		—	
CDR‐SB	DTI‐ALPS					
	With stenosis and low DTI‐ALPS	0.70 (0.47 to 0.94)	0.50 (0.25 to 0.75)	< 0.001	0.42 (0.10 to 0.75)	0.011
	With stenosis and high DTI‐ALPS	0.28 (0.05 to 0.51)	0.08 (−0.17 to 0.32)	0.541	—	
	Without stenosis	0.21 (0.11 to 0.30)	—		—	
	FW‐WM					
	With stenosis and high FW‐WM	0.71 (0.48 to 0.93)	0.50 (0.26 to 0.74)	< 0.001	0.46 (0.12 to 0.80)	0.008
	With stenosis and low FW‐WM	0.24 (−0.01 to 0.50)	0.04 (−0.23 to 0.31)	0.782	—	
	Without stenosis	0.21 (0.11 to 0.30)	—		—	

*Note:* Glymphatic function was assessed using DTI‐ALPS and FW‐WM metrics. Low DTI‐ALPS or high FW‐WM values indicate impaired glymphatic function, while high DTI‐ALPS or low FW‐WM values indicate preserved glymphatic function. All models were adjusted for age, sex, years of education, vascular risk factors (including hypertension, diabetes mellitus, and hyperlipidemia), white matter hyperintensity volume, history of stroke, completeness of the circle of Willis, and scanner model. No adjustment for multiple comparisons was applied.

Abbreviations: CDR‐SB, Clinical Dementia Rating–Sum of Boxes; CI, confidence interval; DTI‐ALPS, diffusion tensor imaging‐analysis along the perivascular space; FW‐WM, free water in white matter; MMSE, Mini‐Mental State Examination.

To assess cognitive worsening, an increase of 1.5 points in CDR‐SB was defined as the event of interest. Kaplan–Meier analyses demonstrated that both stenosis‐low DTI‐ALPS and stenosis‐high FW‐WM were associated with a shorter time to cognitive worsening compared with those without stenosis and those with stenosis but preserved glymphatic function (Figure 6). In Cox proportional hazards models adjusted for age, sex, years of education, scanner model, and baseline CDR‐SB, participants with cerebral artery stenosis and low DTI‐ALPS had a significantly higher risk of cognitive worsening compared to those with stenosis and high DTI‐ALPS (HR = 2.96; 95% CI 1.07–8.19; *p* = 0.036) and compared with participants without stenosis (HR = 4.70; 95% CI 2.36–9.36; *p* < 0.001). For FW‐WM, participants with cerebral artery stenosis and high FW‐WM did not show a significantly increased risk compared with those with stenosis and low FW‐WM (HR = 1.35; 95% CI 0.49–3.67; *p* = 0.561), but had a markedly higher risk compared with participants without stenosis (HR = 3.27; 95% CI 1.65–6.50; *p* = 0.001).

**FIGURE 6:**
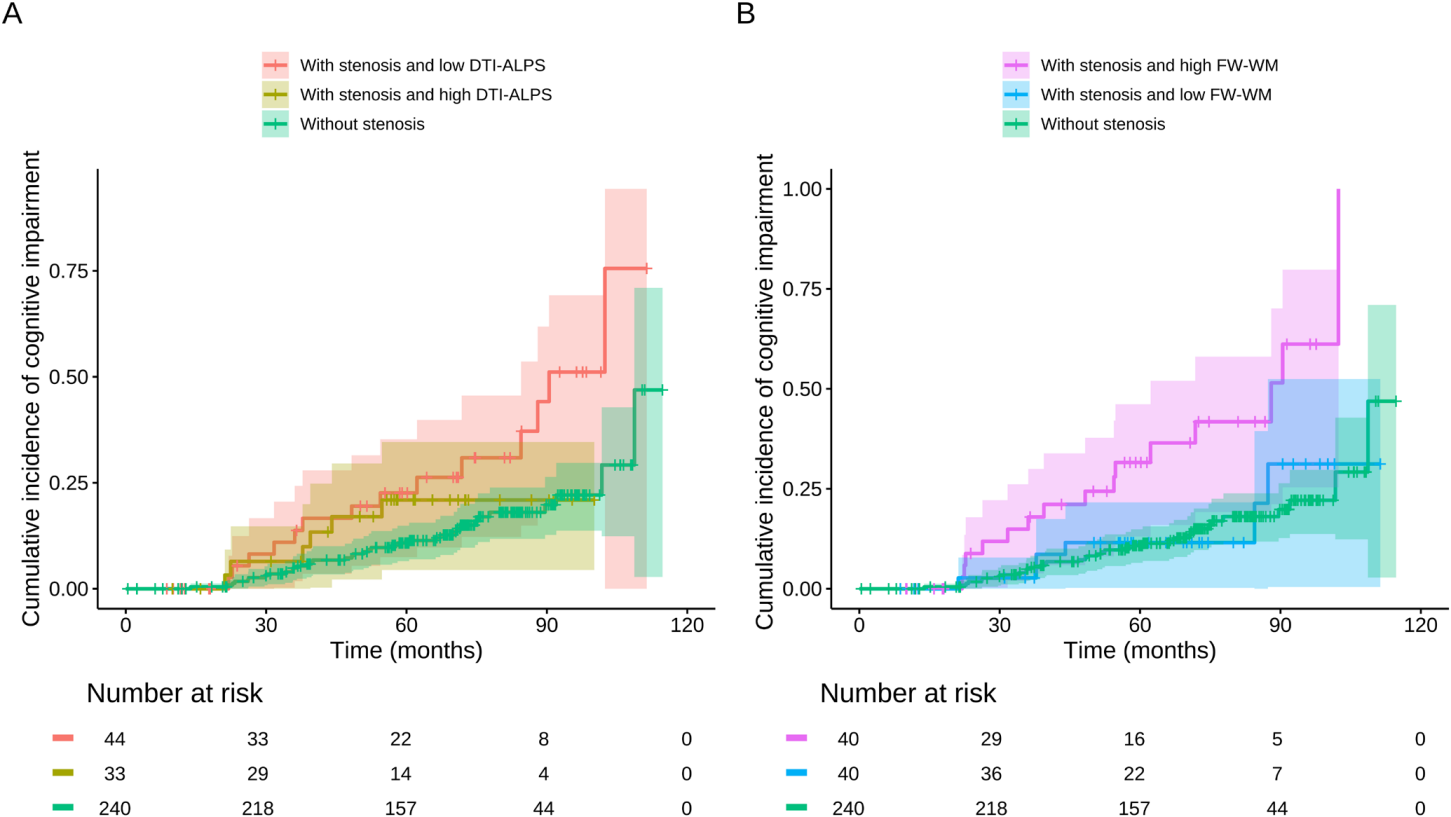
Associations of glymphatic function with risk of cognitive worsening. Kaplan–Meier curves depicting time to cognitive worsening, defined as an increase of 1.5 points in CDR‐SB, stratified by cerebral artery stenosis and glymphatic function. (A) Stratification by cerebral artery stenosis and DTI‐ALPS. (B) Stratification by cerebral artery stenosis and FW‐WM. The tick marks indicate censored cases. Shaded regions represent 95% CIs. CDR‐SB, Clinical Dementia Rating–Sum of Boxes; DTI‐ALPS, diffusion tensor imaging–analysis along the perivascular space; FW‐WM, free water in white matter.

### 3.6 Sensitivity and Subgroup Analyses

In our sensitivity analysis, using a cutoff of ≥ 50% yielded results that were largely consistent in direction and magnitude with the main analysis (Table S4). Among participants without extracranial stenosis, the association of intracranial stenosis and glymphatic dysfunction with cognitive decline remained consistent with the main analysis in both direction and magnitude, with the coexistence of intracranial stenosis and glymphatic dysfunction being associated with the greatest cognitive decline (Table S5). In contrast, among participants without intracranial stenosis, no such association was observed (Table S6).

### 3.7 Associations of DTI‐ALPS With Anterior and Posterior Circulation Stenosis Burdens

DTI‐ALPS showed negative correlations with anterior circulation stenosis burden (*r* = −0.104) and posterior circulation stenosis burden (*r* = −0.173). The difference between the correlation coefficients, calculated as anterior minus posterior, was not statistically significant (Δ*r* = 0.069, 95% CI −0.046 to 0.184, *p* = 0.241).

## 4 Discussion

Our main findings were as follows. First, cerebral artery stenosis was associated with impaired glymphatic function, measured by DTI‐ALPS and FW‐WM. Second, glymphatic dysfunction may represent a significant contributing factor connecting cerebral artery stenosis with cognitive impairment, with individuals showing both cerebral artery stenosis and glymphatic dysfunction experiencing accelerated longitudinal cognitive decline and a markedly higher risk of cognitive worsening. Third, in the association between cerebral artery stenosis and cognitive performance, glymphatic dysfunction, but not Aβ burden, mediated the association. These results suggest that glymphatic dysfunction may be an important downstream pathway linking cerebral artery stenosis to cognitive deterioration.

A central contribution of our study lies in establishing a link between cerebral artery stenosis and perivascular clearance, a mechanism that has remained relatively underexplored. Previous studies have identified multiple determinants of glymphatic function, including sleep, dietary patterns, chronic stress, hypertension, physical activity, and neurodegenerative diseases [37]. However, direct evidence connecting large‐vessel atherosclerotic pathology to glymphatic impairment remains limited. An earlier investigation demonstrated associations between intima‐media thickness in the bilateral common carotid arteries, carotid bulb, and bifurcations and the DTI‐ALPS index [38]. More recently, a study of patients with unilateral intracranial arterial stenosis reported greater glymphatic dysfunction on the stenotic side than on the contralateral side, based on a single DTI‐ALPS metric [39]. Extending these observations, our analysis incorporated two complementary glymphatic markers, DTI‐ALPS and FW‐WM, providing evidence that cerebral arterial stenosis was associated with glymphatic dysfunction. We further identified a pattern consistent with a dose–response relationship, whereby increasing stenosis burden was associated with progressively worse glymphatic indices, which is consistent with a biologically plausible gradient effect. A plausible mechanistic basis for these findings has been proposed in the literature. Glymphatic transport is driven by arterial wall pulsatility, which facilitates the convective influx of cerebrospinal fluid into the brain parenchyma along perivascular spaces [11, 12, 13]. Large‐vessel atherosclerosis is known to reduce arterial compliance, alter pulsatility, and dampen the transmission of pulse pressure waves to distal small vessels, thereby disrupting downstream microvascular hemodynamics [40]. Consequently, stenosis‐induced impairments in pulsatility and microvascular function may reduce the kinetic energy necessary for efficient glymphatic transport, offering one possible physiological link between cerebral artery stenosis and perivascular clearance failure.

Consistent with prior studies, we observed that cerebral artery stenosis was associated with worse cognitive performance at baseline [3, 4, 5, 6]. Traditionally, this association has been attributed primarily to chronic cerebral hypoperfusion and cumulative ischemic injury. Our mediation analyses further suggest that glymphatic dysfunction may serve as a significant contributing factor linking cerebral artery stenosis to cognitive impairment. A major strength of our study is the longitudinal characterization of cognitive trajectories. We found that individuals with both cerebral artery stenosis and impaired glymphatic function experienced more rapid cognitive decline over time and a markedly increased risk of cognitive worsening compared with those who had cerebral artery stenosis but preserved glymphatic function. Another finding of this study is that intracranial, rather than extracranial, arterial stenosis in combination with glymphatic dysfunction was associated with the greatest cognitive decline. These findings indicate that the associations observed in the main analysis were driven predominantly by intracranial rather than extracranial arterial stenosis. This difference may be explained by the closer anatomical proximity of intracranial arteries to the brain parenchyma [7]. Previous studies of intracranial atherosclerotic disease‐related cognitive impairment have primarily focused on stenosis severity, lesion location, or plaque burden as predictors of cognitive outcomes [4, 6]. Adding to existing evidence, our findings suggest that DTI‐ALPS and FW‐WM may serve as novel tools for cognitive risk stratification and could help identify patients who may benefit from targeted interventions aimed at preserving cognitive function.

Cerebral atherosclerosis and AD frequently coexist. Autopsy studies have consistently demonstrated a substantial burden of vascular pathology in individuals with AD [15, 16]. In our cohort, participants with cerebral artery stenosis and concomitant glymphatic dysfunction were older and more likely to carry a clinical diagnosis of AD, supporting a close interaction between vascular and neurodegenerative processes. Accumulating evidence implicates the glymphatic system as an early pathway in AD pathophysiology, potentially occurring before detectable Aβ accumulation [15, 16]. This raises the possibility that vascular disease may facilitate Aβ accumulation through impaired glymphatic clearance, thereby contributing to cognitive decline. To disentangle these interrelated mechanisms, we conducted sensitivity analyses adjusting for Aβ burden and performed multiple mediation analyses incorporating both glymphatic markers and Aβ PET measures. Notably, glymphatic dysfunction emerged as the predominant indirect pathway linking cerebral artery stenosis to cognitive impairment, whereas Aβ burden accounted for a relatively smaller proportion of the mediated effect. Together, these findings suggest that, in the context of cerebral artery stenotic disease, glymphatic impairment may exert a more immediate influence on cognitive function than Aβ pathology.

Strengths of our study include the use of a large, well‐phenotyped longitudinal cohort with dual glymphatic biomarkers and Aβ PET. However, limitations exist. First, DTI‐ALPS and FW‐WM are indirect imaging markers of glymphatic function and do not capture the full complexity of perivascular fluid dynamics. For example, DTI‐ALPS may be confounded by age‐related brain atrophy, white matter hyperintensities, and local cerebrospinal fluid contamination. We also did not evaluate the relationship between arterial stenosis in different cerebral vascular territories and regional glymphatic dysfunction. Future studies incorporating advanced cerebrospinal fluid flow imaging or invasive tracers are needed to validate our findings. Second, the OASIS‐3 cohort is predominantly composed of highly educated White individuals, which may limit generalizability to more diverse populations. For instance, East Asian populations tend to exhibit a higher prevalence of intracranial atherosclerosis, whereas extracranial arterial stenosis is less frequent [22]. Third, the assessment of extracranial arterial disease was incomplete, as TOF‐MRA does not fully capture the proximal internal carotid artery or bifurcation. Finally, TOF‐MRA is susceptible to artifacts, which may result in an overestimation of the degree of luminal narrowing. Future studies incorporating more definitive imaging modalities, such as computed tomography angiography or the gold‐standard digital subtraction angiography, are needed to further validate our conclusions. Moreover, the etiology of stenosis was not formally screened. Given that different vasculopathies may have distinct effects on vessel wall stiffness and pulsatility, they may differentially influence glymphatic function. Future studies incorporating vessel wall imaging are warranted to clarify the potential role of vascular etiology in glymphatic dysfunction.

In summary, our study provides evidence that cerebral artery stenosis is associated with impaired glymphatic function and suggests that glymphatic dysfunction, rather than Aβ burden, may partially mediate the relationship between cerebral artery stenosis and cognitive impairment. These results suggest that the glymphatic system may warrant further investigation as a potential imaging biomarker and candidate therapeutic target for cognitive impairment in cerebral artery stenosis.

## Funding

This study was supported by grants from the Young Scientists Fund of the National Natural Science Foundation of China (82602521), Guangdong Provincial Clinical Research Center for Neurological Diseases (2020B1111170002), Guangdong Province International Cooperation Base for Early Intervention and Functional Rehabilitation of Neurological Diseases (2020A0505020004), Guangzhou Major Difficult and Rare Diseases Project (2024MDRD02), China Postdoctoral Science Foundation (2025M782381), Guangdong Provincial Engineering Center for Major Neurological Disease Treatment, Guangdong Provincial Translational Medicine Innovation Platform for Diagnosis and Treatment of Major Neurological Disease, Guangzhou Clinical Research and Translational Center for Major Neurological Diseases.

## Ethics Statement

The original study was approved by the Institutional Review Board of Washington University School of Medicine (No. 201109148).

## Consent

Written informed consent was obtained from all participants in accordance with the Declaration of Helsinki.

## Conflicts of Interest

The authors declare no conflicts of interest.

## Supporting information


**Table S1:** Characteristics of included and excluded participants in the study cohort.
**Table S2:** Characteristics of the study cohort.
**Table S3:** Baseline characteristics of the study cohort stratified by cerebral artery stenosis and glymphatic function.
**Table S4:** Sensitivity analysis of longitudinal cognitive decline by cerebral artery stenosis and glymphatic function using a 50% stenosis cutoff.
**Table S5:** Sensitivity analysis of longitudinal cognitive decline by intracranial cerebral artery stenosis and glymphatic function in participants without extracranial arterial stenosis.
**Table S6:** Sensitivity analysis of longitudinal cognitive decline by extracranial artery stenosis and glymphatic function in participants without intracranial arterial stenosis.
**Figure S1:** Examples of normal and stenotic cerebral vessels.
**Figure S2:** Patient inclusion flowchart.
**Figure S3:** Glymphatic function according to the degree of cerebral artery stenosis.
**Figure S4:** Mediation of the association between cerebral artery stenosis and cognitive performance by glymphatic function at baseline.

## Data Availability

The data that support the findings of this study are available in OASIS‐3 open‐access data at https://www.oasis‐brains.org. These data were derived from the following resources available in the public domain: OASIS‐3 open‐access data, https://www.oasis‐brains.org.
