# Supplementary material for: Cerebral Artery Stenosis, Glymphatic Dysfunction, and Cognitive Trajectories: A Longitudinal Cohort Study of Community‐Dwelling Older Adults
Source: CNS Neurosci Ther. 2026 Oct 21;32(10):e71216. doi: 10.1002/cns.71216 (PMC13685670; doi:10.1002/cns.71216)
Supplement: Supplementary file 1 [file CNS-32-e71216-s001.pdf]

SUPPLEMENTARY MATERIAL

Cerebral Artery Stenosis, Glymphatic Dysfunction, and Cognitive Trajectories:

A Longitudinal Cohort Study of Community-Dwelling Older Adults

TABLE OF CONTENTS

Table S1. Characteristics of included and excluded participants in the study cohort 2

Table S2. Characteristics of the study cohort 4

Table S3. Baseline characteristics of the study cohort stratified by cerebral artery stenosis and glymphatic function 6

Table S4. Sensitivity analysis of longitudinal cognitive decline by cerebral artery stenosis and glymphatic function using a 50% stenosis cutoff 8

Table S5. Sensitivity analysis of longitudinal cognitive decline by intracranial cerebral artery stenosis and glymphatic function in participants without extracranial arterial stenosis 10

Table S6. Sensitivity analysis of longitudinal cognitive decline by extracranial artery stenosis and glymphatic function in participants without intracranial arterial stenosis 12

Figure S1. Examples of normal and stenotic cerebral vessels. 14

Figure S2. Patient inclusion flowchart 15

Figure S3. Glymphatic function according to the degree of cerebral artery stenosis. 16

Figure S4. Mediation of the association between cerebral artery stenosis and cognitive performance by glymphatic function at baseline 17

Table S1. Characteristics of included and excluded participants in the study cohort

	Included (n=346)	Excluded (n=1032)
Age, years	66.40 ± 9.05	69.95 ± 8.98
Female	189 (54.6%)	567 (54.9%)
Education years	16 [14, 18]	16 [14, 18]
Race		
Asian	1 (0.3%)	7 (0.7%)
Black	51 (14.7%)	158 (15.3%)
Mix or others	0 (0%)	10 (1.0%)
White	294 (85.0%)	857 (83.0%)
ApoE		
ε2/ε2	3 (0.9%)	5 (0.5%)
ε2/ε3	39 (11.3%)	104 (10.2%)
ε2/ε4	9 (2.6%)	37 (3.6%)
ε3/ε3	171 (49.7%)	476 (46.5%)
ε3/ε4	107 (31.1%)	338 (33.0%)
ε4/ε4	15 (4.4%)	63 (6.2%)
Disease and lifestyle at baseline and during all follow-up visits		
Stroke or TIA	15 (4.3%)	104 (10.1%)
Alzheimer's disease	60 (17.3%)	204 (19.8%)
Hypertension	181 (52.3%)	555 (53.8%)
Hypercholesterolemia	182 (52.6%)	588 (57.0%)
Diabetes mellitus	37 (10.7%)	129 (12.5%)
Smoker	29 (8.4%)	80 (7.8%)

Alcohol	26 (7.5%)	52 (5.0%)

Data are presented as mean ± standard deviation or n (%) or median [interquartile range]. Abbreviations: TIA indicates transient ischemic attack.

Table S2. Characteristics of the study cohort

	Overall (n=346)
Age, years	66.40 ± 9.05
Female	189 (54.6%)
Education years	16 [14, 18]
Race	
Asian	1 (0.3%)
Black	51 (14.7%)
White	294 (85.0%)
Right-hand dominant	317 (92.7%)
ApoE	
ε2/ε2	3 (0.9%)
ε2/ε3	39 (11.3%)
ε2/ε4	9 (2.6%)
ε3/ε3	171 (49.7%)
ε3/ε4	107 (31.1%)
ε4/ε4	15 (4.4%)
History of disease and lifestyle	
Stroke or TIA	15 (4.3%)
Alzheimer's disease	60 (17.3%)
Hypertension	174 (51.2%)
Hypercholesterolemia	177 (52.1%)
Diabetes mellitus	33 (9.7%)
Smoker	19 (5.6%)
Alcohol	22 (6.5%)
Cerebral artery stenosis *	
Extracranial internal carotid artery	32 (9.2%)
Intracranial internal carotid artery	2 (0.6%)

Middle cerebral artery	23 (6.6%)
Anterior cerebral artery	13 (3.8%)
Posterior cerebral artery	38 (11.0%)
Vertebral artery	14 (4.0%)
Basilar artery	7 (2.0%)
Completion of cognitive assessment	
MMSE	334 (96.5%)
CDR-SB	335 (96.8%)
Baseline cognitive assessment to imaging interval, days	
MMSE	-32 [-66, 257.25]
CDR-SB	-32 [-66, 259]
Baseline cognitive assessment score	
MMSE	29 [28, 30]
CDR-SB	0 [0, 0]
Last follow-up cognitive assessment to imaging interval, days	
MMSE	1457.5 [1006.75, 2076.5]
CDR-SB	2034 [1476.25, 2483.25]
Last follow-up cognitive assessment score	
MMSE	29 [28, 30]
CDR-SB	0 [0, 0.12]

Data are presented as mean ± standard deviation or n (%) or median [interquartile range]. Abbreviations: DTI-ALPS, diffusion tensor imaging-analysis along the perivascular space; FW-WM, free water in white matter; MMSE, Mini-Mental State Examination; CDR-SB, Clinical Dementia Rating–Sum of Boxes.

Missing data were observed for hand dominant (n = 4), APOE (n = 2), hypertension (n = 6), hypercholesterolemia (n = 6), diabetes mellitus (n = 6), smoker (n = 5), alcohol (n = 5).

* Each arterial segment is combined for the left and/or right sides.

Table S3. Baseline characteristics of the study cohort stratified by cerebral artery stenosis and glymphatic function

	DTI-ALPS (n=346)				FW-WM (n=342) *			
	Without stenosis (n=259)	With stenosis and low DTI-ALPS (n=47)	With stenosis and high DTI-ALPS (n=40)	p value	Without stenosis (n=259)	With stenosis and low FW-WM (n=36)	With stenosis and high FW-WM (n=47)	p value
Age, years	65.63 ± 9.11	71.72 ± 7.60	65.12 ± 8.27	<0.001	65.63 ± 9.11	66.72 ± 7.74	69.65 ± 9.02	0.018
Female	139 (53.7%)	26 (55.3%)	24 (60.0%)	0.752	139 (53.7%)	22 (61.1%)	25 (53.2%)	0.692
Education years	16 [14, 18]	16 [12, 18]	16 [14.75, 18]	0.142	16 [14, 18]	16 [14, 18]	16 [12, 18]	0.239
History of stroke or TIA	11 (4.2%)	1 (2.1%)	3 (7.5%)	0.467	11 (4.2%)	2 (5.6%)	2 (4.3%)	0.936
History of Alzheimer's disease	39 (15.1%)	16 (34.0%)	5 (12.5%)	0.005	39 (15.1%)	6 (16.7%)	14 (29.8%)	0.048

Hypertension	128 (50.4%)	26 (56.5%)	20 (50.0%)	0.737	128 (50.4%)	16 (44.4%)	28 (60.9%)	0.294
Hypercholesterolemia	131 (51.6%)	29 (63.0%)	17 (42.5%)	0.156	131 (51.6%)	20 (55.6%)	23 (50.0%)	0.875
Diabetes mellitus	24 (9.4%)	6 (13.0%)	3 (7.5%)	0.662	24 (9.4%)	2 (5.6%)	7 (15.2%)	0.318
Baseline MMSE	29 [28.5, 30]	28 [27, 29.5]	30 [29, 30]	<0.001	29 [28.5, 30]	29.5 [28.25, 30]	29 [27, 30]	0.071
Baseline CDR-SB	0 [0, 0]	0 [0, 0.75]	0 [0, 0]	0.026	0 [0, 0]	0 [0, 0]	0 [0, 1.50]	0.032

Participants without cerebral artery stenosis served as a shared reference group. Among participants with stenosis, glymphatic function was separately stratified using DTI-ALPS and FW-WM metrics, resulting in two parallel three-group stratifications. The "without stenosis" group is identical in both stratifications and is presented twice for clarity.

Data are presented as mean ± SD or n (%) or median [interquartile range].

Abbreviations: DTI-ALPS, diffusion tensor imaging-analysis along the perivascular space; FW-WM, free water in white matter; MMSE, Mini-Mental State Examination; CDR-SB, Clinical Dementia Rating–Sum of Boxes; TIA: transient ischemic attack.

Missing data were observed for hypertension (n = 6), hypercholesterolemia (n = 6), diabetes mellitus (n = 6).

* 4 without available FW-WM in stenosis group.

Table S4. Sensitivity analysis of longitudinal cognitive decline by cerebral artery stenosis and glymphatic function using a 50% stenosis cutoff

Glymphatic function marker	Cognitive measure (slope difference, points/year, unless noted)	Stenosis with impaired GF vs. Without stenosis	Stenosis with impaired GF vs. Stenosis with preserved GF	Stenosis with preserved GF vs. Without stenosis
DTI-ALPS	MMSE [a]	-0.287 (-0.656 to 0.082)	-0.048 (-0.569 to 0.472)	-0.239 (-0.622 to 0.144)
	CDR-SB [a]	0.947 (0.602 to 1.293)	0.870 (0.385 to 1.356)	0.077 (-0.282 to 0.437)
	Cognitive worsening (HR) [b]	4.22 (1.64 to 10.85)	2.56 (0.39 to 16.68)	1.65 (0.29 to 9.30)
FW-WM	MMSE [a]	-0.255 (-0.567 to 0.057)	0.055 (-0.537 to 0.646)	-0.310 (-0.824 to 0.205)
	CDR-SB [a]	0.648 (0.334 to 0.962)	0.364 (-0.159 to 0.887)	0.284 (-0.151 to 0.719)
	Cognitive worsening (HR) [b]	3.47 (1.28 to 9.37)	1.25 (0.24 to 6.45)	2.77 (0.65 to 11.75)

[a] Longitudinal cognitive trajectories were analyzed using linear mixed-effects models with random intercepts and slopes to account for repeated measures within participants. Fixed effects included baseline age, sex, years of education, vascular risk factors (hypertension, diabetes mellitus, and hyperlipidemia), history of stroke, white matter hyperintensity volume, completeness of the circle of Willis, scanner model, cerebral artery stenosis status, glymphatic function group, and their interactions with time. The primary outcomes were the estimated mean trajectories of

MMSE and CDR-SB scores over time. Differences in slope are calculated as the former group minus the latter group. Negative differences indicate faster MMSE decline, whereas positive differences indicate faster CDR-SB worsening in the former group.

[b] Cox proportional hazards models were used to estimate HRs and 95% CIs for cognitive worsening, adjusting for baseline age, sex, years of education, scanner model, and baseline cognitive score. Cognitive worsening was defined as an increase of ≥1.5 points in CDR-SB from baseline.

All values in parentheses represent 95% confidence intervals.

Abbreviations: DTI-ALPS, diffusion tensor imaging-analysis along the perivascular space; FW-WM, free water in white matter; MMSE, Mini-Mental State Examination; CDR-SB, Clinical Dementia Rating–Sum of Boxes; HR, hazard ratio; CI, confidence interval; GF, glymphatic function.

Table S5. Sensitivity analysis of longitudinal cognitive decline by intracranial cerebral artery stenosis and glymphatic function in participants without extracranial arterial stenosis

Glymphatic function marker	Cognitive measure (slope difference, points/year, unless noted)	Stenosis with impaired GF vs. Without stenosis	Stenosis with impaired GF vs. Stenosis with preserved GF	Stenosis with preserved GF vs. Without stenosis
DTI-ALPS	MMSE [a]	-0.432 (-0.717 to -0.147)	-0.157 (-0.544 to 0.231)	-0.275 (-0.563 to 0.012)
	CDR-SB [a]	0.603 (0.320 to 0.886)	0.587 (0.185 to 0.989)	0.016 (-0.296 to 0.327)
	Cognitive worsening (HR) [b]	5.79 (2.44 to 13.73)	4.80 (0.98 to 23.54)	1.21 (0.28 to 5.21)
FW-WM	MMSE [a]	-0.382 (-0.628 to -0.137)	-0.093 (-0.518 to 0.332)	-0.289 (-0.654 to 0.076)
	CDR-SB [a]	0.412 (0.142 to 0.682)	0.213 (-0.210 to 0.636)	0.199 (-0.150 to 0.549)
	Cognitive worsening (HR) [b]	3.91 (1.64 to 9.28)	1.66 (0.40 to 6.88)	2.36 (0.64 to 8.62)

[a] Longitudinal cognitive trajectories were analyzed using linear mixed-effects models with random intercepts and slopes to account for repeated measures within participants. Fixed effects included baseline age, sex, years of education, vascular risk factors (hypertension, diabetes mellitus, and hyperlipidemia), history of stroke, white matter hyperintensity volume, completeness of the circle of Willis, scanner model, cerebral artery stenosis status, glymphatic function group, and their interactions with time. The primary outcomes were the estimated mean trajectories of

MMSE and CDR-SB scores over time. Differences in slope are calculated as the former group minus the latter group. Negative differences indicate faster MMSE decline, whereas positive differences indicate faster CDR-SB worsening in the former group.

[b] Cox proportional hazards models were used to estimate HRs and 95% CIs for cognitive worsening, adjusting for baseline age, sex, years of education, scanner model, and baseline cognitive score. Cognitive worsening was defined as an increase of ≥1.5 points in CDR-SB from baseline.

All values in parentheses represent 95% confidence intervals.

Abbreviations: DTI-ALPS, diffusion tensor imaging-analysis along the perivascular space; FW-WM, free water in white matter; MMSE, Mini-Mental State Examination; CDR-SB, Clinical Dementia Rating–Sum of Boxes; HR, hazard ratio; CI, confidence interval; GF, glymphatic function.

Table S6. Sensitivity analysis of longitudinal cognitive decline by extracranial artery stenosis and glymphatic function in participants without intracranial arterial stenosis

Glymphatic function marker	Cognitive measure (slope difference, points/year, unless noted)	Stenosis with impaired GF vs. Without stenosis	Stenosis with impaired GF vs. Stenosis with preserved GF	Stenosis with preserved GF vs. Without stenosis
DTI-ALPS	MMSE [a]	0.192 (-0.247 to 0.631)	0.114 (-0.431 to 0.659)	0.078 (-0.260 to 0.416)
	CDR-SB [a]	-0.197 (-0.701 to 0.308)	-0.320 (-0.946 to 0.306)	0.124 (-0.265 to 0.512)
	Cognitive worsening (HR) [b]	-*	-*	2.64 (0.45 to 15.54)
FW-WM	MMSE [a]	0.196 (-0.204 to 0.597)	0.144 (-0.387 to 0.674)	0.053 (-0.308 to 0.414)
	CDR-SB [a]	0.327 (-0.163 to 0.818)	0.482 (-0.139 to 1.104)	-0.155 (-0.553 to 0.243)
	Cognitive worsening (HR) [b]	0.97 (0.08 to 11.33)	0.37 (0.01 to 11.84)	2.61 (0.22 to 31.49)

[a] Longitudinal cognitive trajectories were analyzed using linear mixed-effects models with random intercepts and slopes to account for repeated measures within participants. Fixed effects included baseline age, sex, years of education, vascular risk factors (hypertension, diabetes mellitus, and hyperlipidemia), history of stroke, white matter hyperintensity volume, completeness of the circle of Willis, scanner model, cerebral artery stenosis status, glymphatic function group, and their interactions with time. The primary outcomes were the estimated mean trajectories of

MMSE and CDR-SB scores over time. Differences in slope are calculated as the former group minus the latter group. Negative differences indicate faster MMSE decline, whereas positive differences indicate faster CDR-SB worsening in the former group.

[b] Cox proportional hazards models were used to estimate HRs and 95% CIs for cognitive worsening, adjusting for baseline age, sex, years of education, scanner model, and baseline cognitive score. Cognitive worsening was defined as an increase of ≥1.5 points in CDR-SB from baseline.

* HR could not be estimated because the number of participants was insufficient.

All values in parentheses represent 95% confidence intervals.

Abbreviations: DTI-ALPS, diffusion tensor imaging-analysis along the perivascular space; FW-WM, free water in white matter; MMSE, Mini-Mental State Examination; CDR-SB, Clinical Dementia Rating–Sum of Boxes; HR, hazard ratio; CI, confidence interval; GF, glymphatic function.

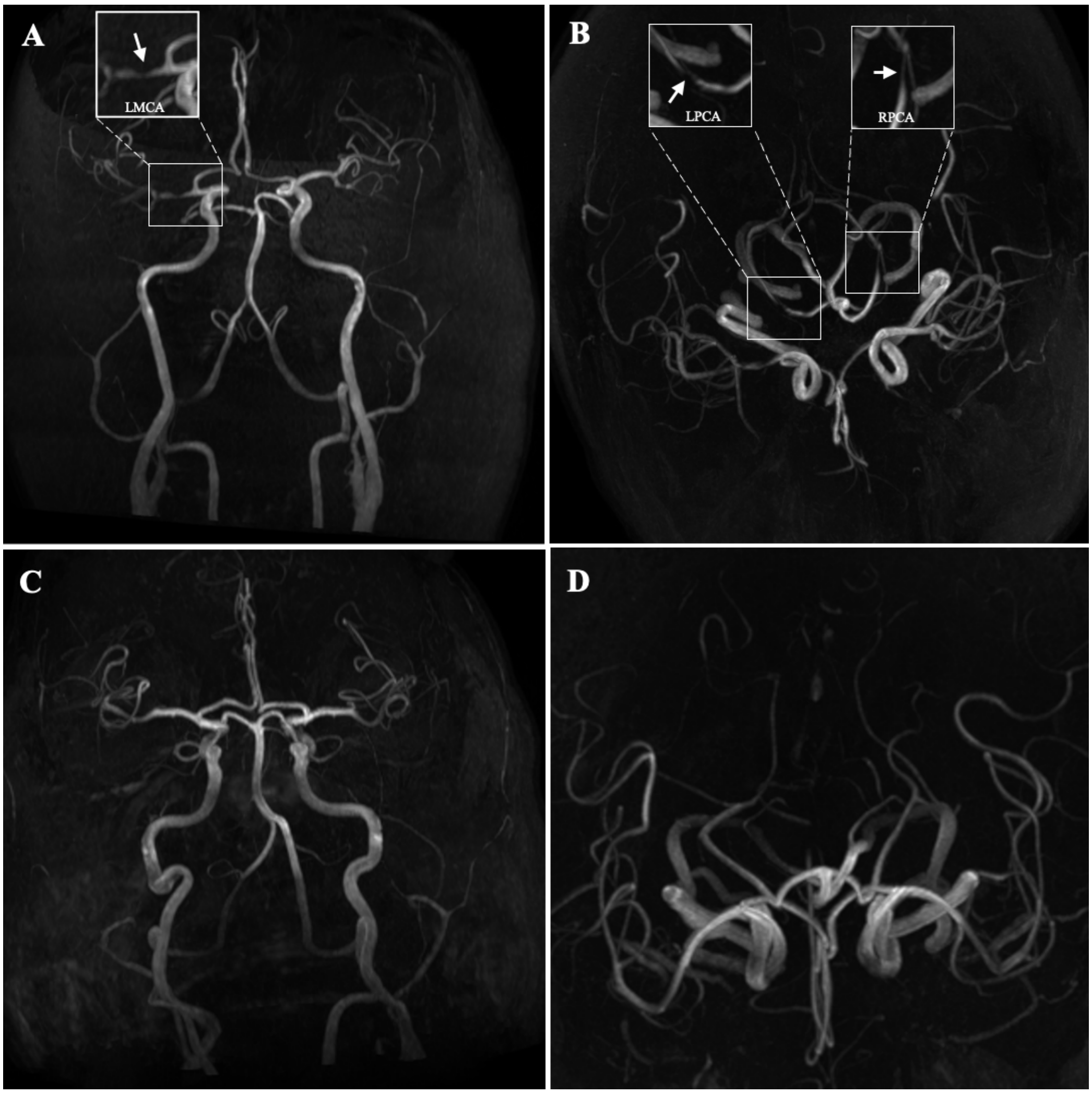

Figure S1. Examples of normal and stenotic cerebral vessels.

Maximum intensity projection images derived from time-of-flight magnetic resonance angiography are shown. Patient 1 (A-B) was an 84-year-old woman with vascular stenosis involving the left middle cerebral artery (arrow in A) and bilateral posterior cerebral arteries (arrows in B). She had a stenosis burden score of 3, a diffusion tensor image analysis along the perivascular space (DTI-ALPS) index of 0.88, and a free water in white matter (FW-WM) value of 0.27, and was classified as having glymphatic dysfunction. Patient 2 was a 58-year-old man without intracranial vascular stenosis (C–D). His stenosis burden score was 0, with an DTI-ALPS index of 1.38 and an FW-WM value of 0.18, and he was classified as having normal glymphatic function.

Abbreviations: LMCA indicates left middle cerebral artery; RPCA, right posterior cerebral artery; LPCA, left posterior cerebral artery.

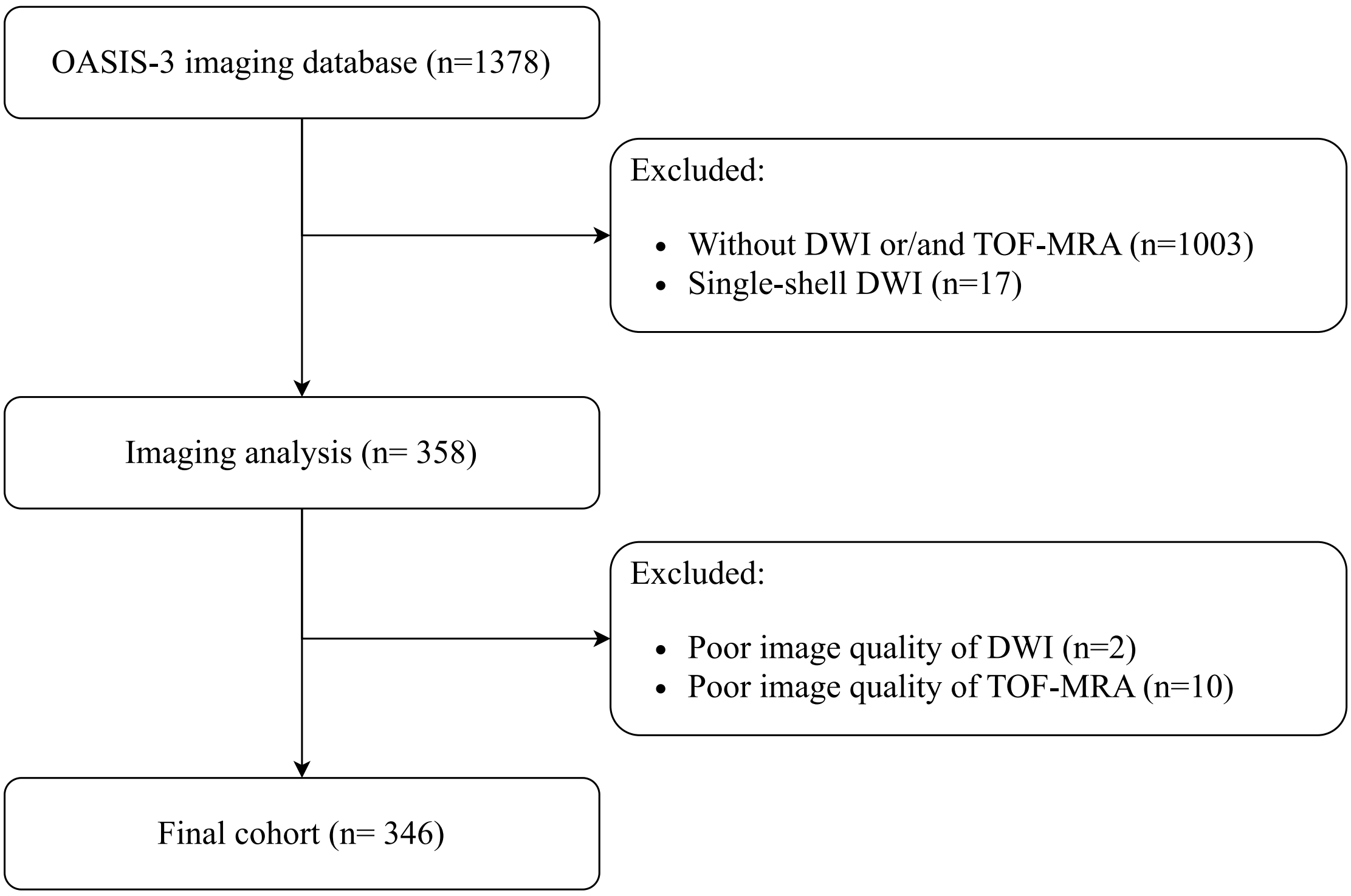

Figure S2. Patient inclusion flowchart

Abbreviations: OASIS-3, Open Access Series of Imaging Studies-3; DWI, diffusion-weighted imaging; TOF-MRA, time-of-flight magnetic resonance angiography.

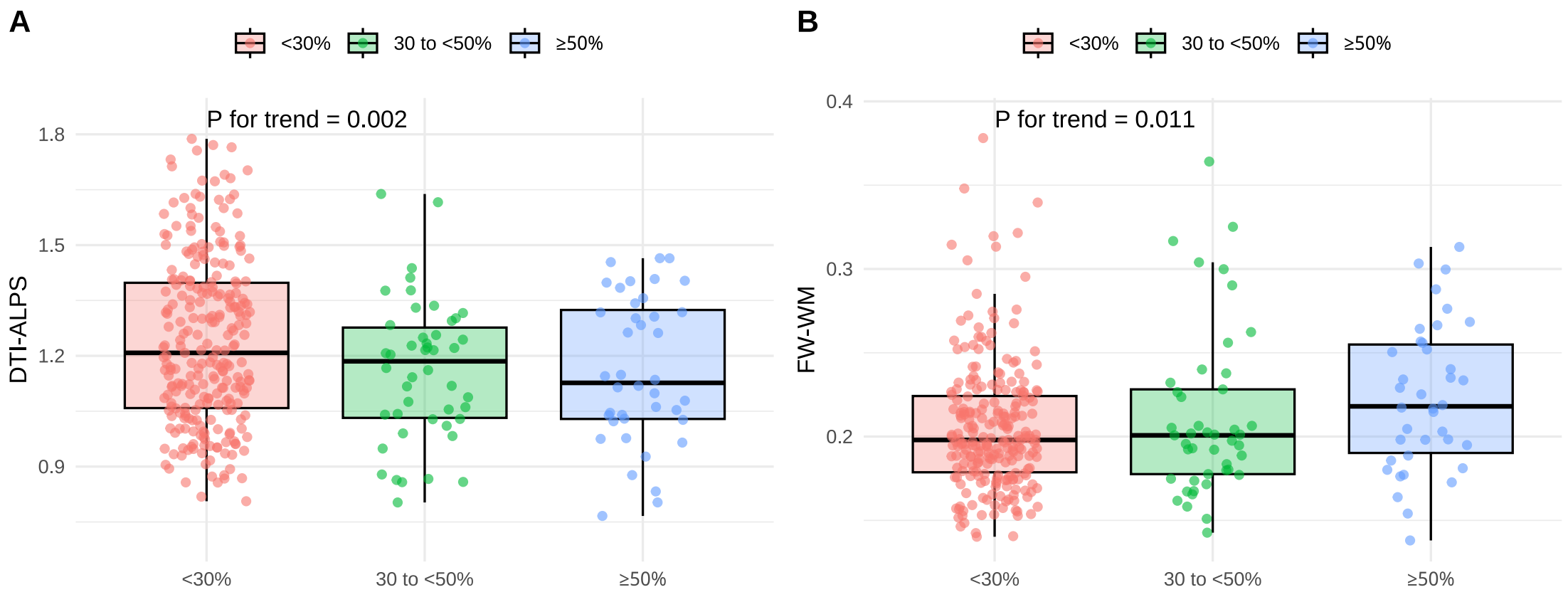

Figure S3. Glymphatic function according to the degree of cerebral artery stenosis.

(A) Boxplots comparing the DTI–ALPS index in participants with 30 to <50% cerebral artery stenosis, ≥50% cerebral artery stenosis, and no cerebral artery stenosis.

(B) Boxplots comparing the FW-WM in participants with 30 to <50% cerebral artery stenosis, ≥50% cerebral artery stenosis, and no cerebral artery stenosis.

Abbreviations: DTI–ALPS, diffusivity along the perivascular space index; FW-WM, white matter free water.

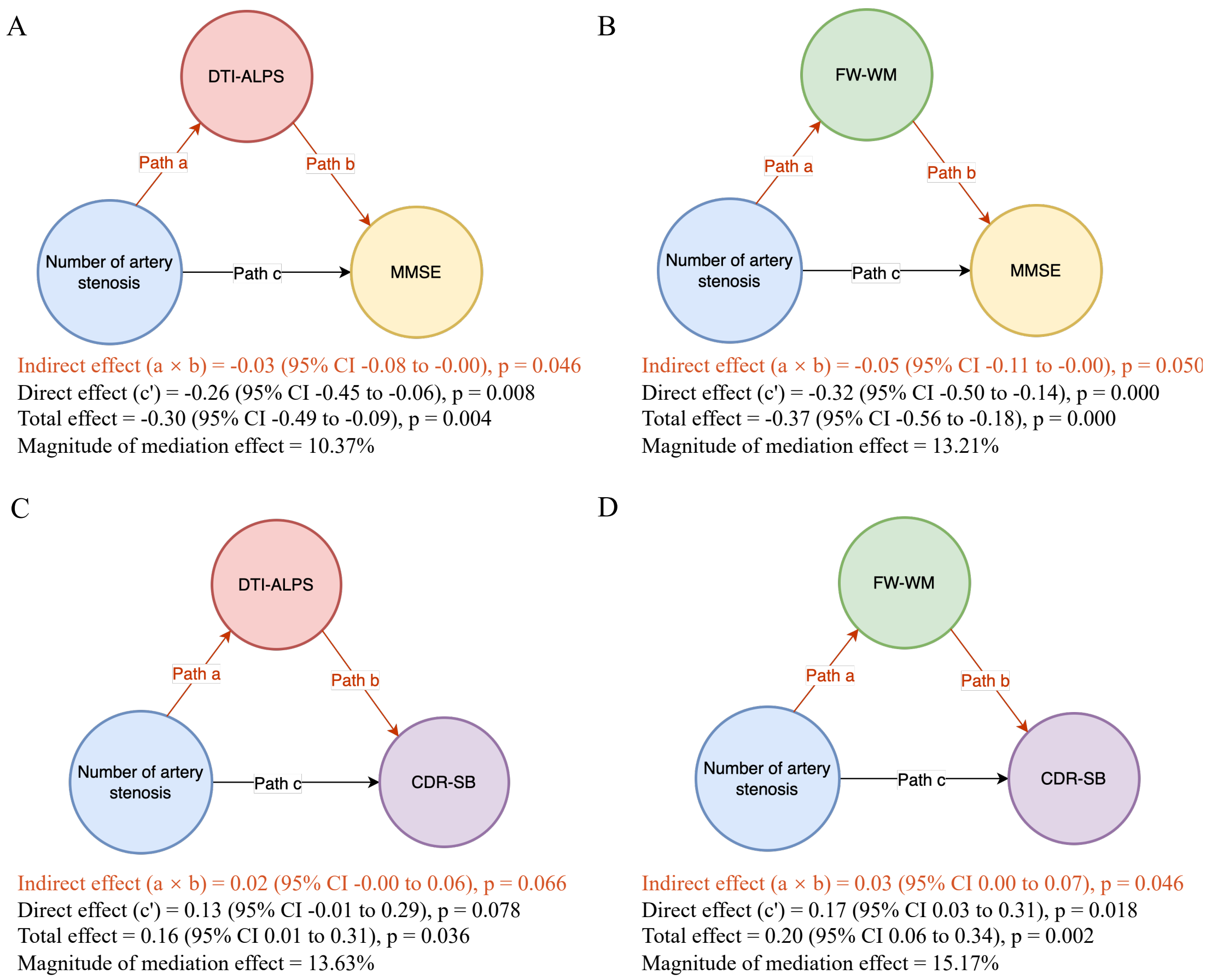

Figure S4. Mediation of the association between cerebral artery stenosis and cognitive performance by glymphatic function at baseline

(A-B) Indirect effect of the number of stenotic arteries on baseline MMSE scores mediated by (A) DTI-ALPS and (B) FW-WM. (C-D) Indirect effect of the number of stenotic arteries on baseline CDR-SB scores mediated by (C) DTI-ALPS and (D) FW-WM. All models were adjusted for age, sex, years of education, the interval between imaging and baseline cognitive assessment, history of stroke, vascular risk factors (including hypertension, diabetes mellitus, and hyperlipidemia), white matter hyperintensity volume, completeness of the circle of Willis, scanner model, and Aβ PET.

Abbreviations: DTI-ALPS, diffusion tensor imaging–analysis along the perivascular space; FW-WM, free water in white matter; MMSE, Mini-Mental State Examination; CDR-SB, Clinical Dementia Rating–Sum of Boxes.
